# Aptamer selection against cell extracts containing the zoonotic obligate intracellular bacterium, *Anaplasma phagocytophilum*

**DOI:** 10.1038/s41598-024-52808-8

**Published:** 2024-01-30

**Authors:** Lisa Lucie Le Dortz, Clotilde Rouxel, Quentin Leroy, Frédéric Ducongé, Henri-Jean Boulouis, Nadia Haddad, Pierre Lucien Deshuillers, Anne-Claire Lagrée

**Affiliations:** 1 Anses, INRAe, Ecole Nationale Vétérinaire d’Alfort, UMR BIPAR, Laboratory of Animal Health, 94700 Maisons-Alfort, France; 2 CEA, Fundamental Research Division (DRF), Institute of Biology François Jacob, Molecular Imaging Research Center, CNRS UMR9199, Paris-Saclay University, 92265 Fontenay-Aux-Roses, France

**Keywords:** Microbiology, Molecular biology, Diseases

## Abstract

*A. phagocytophilum* is a zoonotic and tick-borne bacterium, threatening human and animal health. Many questions persist concerning the variability of strains and the mechanisms governing the interactions with its different hosts. These gaps can be explained by the difficulty to cultivate and study *A. phagocytophilum* because of its strict intracellular location and the lack of specific tools, in particular monoclonal antibodies, currently unavailable. The objective of our study was to develop DNA aptamers against *A. phagocytophilum,* or molecules expressed during the infection, as new study and/or capture tools. Selecting aptamers was a major challenge due to the strict intracellular location of the bacterium. To meet this challenge, we set up a customized selection protocol against an enriched suspension of *A. phagocytophilum* NY18 strain, cultivated in HL-60 cells. The implementation of SELEX allowed the selection of three aptamers, characterized by a high affinity for HL-60 cells infected with *A. phagocytophilum* NY18 strain. Interestingly, the targets of these three aptamers are most likely proteins expressed at different times of infection. The selected aptamers could contribute to increase our understanding of the interactions between *A. phagocytophilum* and its hosts, as well as permit the development of new diagnostic, therapeutic or drug delivery appliances.

## Introduction

*Anaplasma phagocytophilum* is a strict intracellular bacterium, mainly transmitted by hard ticks of the *Ixodes* genus^1^. This bacterium is responsible for granulocytic anaplasmosis and has major economic and clinical impacts for both human and animal health. Human granulocytic anaplasmosis (HGA) is an emerging zoonosis, described throughout North America, Asia and Europe^2^. In the USA, HGA constitutes a public health issue, with a steadily increasing incidence: 1,761 human cases were reported in 2010, while over 5,600 cases were reported in 2019^3^. In this country, 36% of patients require hospitalization and the estimated lethality rate is less than 1%^4^. The number of human cases is probably underestimated since the main clinical manifestations are most commonly mild with a flu-like syndrome: hyperthermia, malaise, myalgia and migraine^2^. A minority of patients present arthralgia, gastrointestinal, respiratory or neurological symptoms. On the contrary, in Europe, the incidence of HGA remains low, estimated under 300 cases and clinical signs are generally less severe, with no reported deaths to date^2^. In Europe still, *A. phagocytophilum* is considered the most widespread tick-borne pathogen in animals and is responsible for granulocytic anaplasmosis in domestic ruminants (sheep, cattle, etc.) while currently, very few cases of granulocytic anaplasmosis in domestic ruminants have been reported in the USA^4,5^. The most common clinical signs associated with *A. phagocytophilum* infection in ruminants are hyperthermia, agalactia, anorexia, abortion, and lameness. *A. phagocytophilum* also exerts immunosuppressive effects by altering neutrophil and lymphocyte functions^6^. This frequently results in secondary infections, notably tick-borne pyaemia caused by *Staphylococcus aureus*, a fatal complication that can affect up to 30% of lambs infected with *A. phagocytophilum*^7^. *A. phagocytophilum* is therefore considered to contribute to important economic losses in cattle and sheep industry^8^. *A. phagocytophilum* is thought to be maintained among mostly or exclusively asymptomatic wild reservoirs, such as rodents or wild ruminants in the USA^9^. In Europe, the epidemiological cycles of this multi-host agent are poorly understood, the reservoirs remain unknown and targeted health management measures are therefore difficult to implement^1^.

In vivo, *A. phagocytophilum* infects mainly neutrophils, and has also been detected in eosinophils and monocytes, as well as in endothelial cells^10^. The bacterium resides within compact inclusions (micro-colonies or *morulae*) in cytoplasmic vacuoles derived from the host cell. Two bacterial morphotypes exist in mammalian and tick cells: a larger reticulate replicative form (RC) and a smaller dense core form (DC) corresponding to the infectious form ^11^. To invade cells and ensure its survival, *A. phagocytophilum* modulates diverse molecular pathways within the host cell, which are responsible for remodeling the cytoskeleton, manipulating the cellular immune response, hijacking metabolic pathways and delaying apoptosis to its benefit^10,12^. However, the mechanisms of host/pathogen interactions, remain largely to be explored, limiting the development of new therapeutic/screening/diagnostic strategies.

These gaps in knowledge can be explained by the difficulty to isolate and cultivate *A. phagocytophilum*, because of its strict intracellular location and its very short survival in blood samples (neutrophils), but also because very few permissive cells are available for in vitro culture. Only one vertebrate model (HL-60 cells) is permissive to human *A. phagocytophilum* strains, while tick cell models can be used to culture all types of strains (from ruminants, humans, rodents and other wildlife and domestic animals)^13^. Currently, very few strains have been isolated worldwide^14–18^ and very few genomes have been sequenced worldwide (33 published genomes, available on https://www.ncbi.nlm.nih.gov/datasets/genome/?taxon=948). Molecular tools to study the bacterium and its molecular interactions in cellular models are very limited and currently, there are no commercially available monoclonal antibodies.

In 1990, two studies carried out by two different teams led to the discovery of aptamers and the description of the selection method^19,20^. Aptamers are oligonucleotides (ssDNA, RNA, chemically modified nucleic acids), generally less than 100 nucleotides, characterized by a unique three-dimensional structure. Their folding allow them to interact with their target with high affinity and specificity, via Van der Walls, electrostatic or hydrogen bonds^21^. Aptamers have been selected against a wide variety of targets, including small compounds, proteins and even viruses, prokaryotic or eukaryotic cells. Since their discovery, there has been growing interest in their use for diagnosis, treatment and bioimaging, in particular in the field of infectious diseases in human/veterinary medicine^22^. Certain aptamers have been developed to control tick-borne diseases. Tabb et al*.* combined aptamers with the SERS (Surface Enhanced Raman Spectroscopy) method for the detection of the OspA protein, which improved the direct detection of the agent responsible for Lyme disease from blood samples ^23^. Aptamers have significant advantages compared to monoclonal antibodies: better stability, non-toxicity and ease to synthesize^24^. Aptamers are obtained by an in vitro selection method, named SELEX for Systematic Evolution of Ligands by Exponential Enrichment^20^. The SELEX process consists of repeated selection cycles, including three steps: incubation with the target, amplification of the bound sequences, and generation of single-stranded DNA/RNA^25^. In the case of DNA aptamers, a ssDNA library (10^14^–10^15^ different sequences) is incubated with the target of interest. Unbound aptamers are washed away while bound oligonucleotides are eluted and then amplified by PCR. Single-stranded DNA is generated from double stranded PCR products to start a new selection cycle.

The selection of aptamers against strict intracellular bacteria is particularly challenging and has been poorly described in the literature^26^. In fact, the purification of intracellular bacteria is complex, and the final suspension unavoidably contains host cell debris, reducing the chances of selecting aptamers specific to the bacterium and not to components of the host cell. To the best of our knowledge, only one study has developed aptamers against strict intracellular bacteria and was performed for the specific detection of *Rickettsia typhi* within the genus *Rickettsia*. In this study, aptamers were selected against *Rickettsia typhi*, purified on density gradient from murine host cells. After selection, three aptamers demonstrated high affinity and were specific to the family *Rickettsiaceae*. These aptamers were further used for an Enzyme-Linked Fluorescent DNA Aptamer-Magnetic Bead Sandwich assay, which was not able to discriminate between the species of *Rickettsia*, despite the negative selections performed on *R. bellii*, *R. conorii*, *R. parkeri*, and *R. rickettsii* during the selection process. Although *A. phagocytophilum* belongs to the same order (but not the same family) as the genus of *Rickettsia*, these aptamers were not able to bind *A. phagocytophilum* in our hands (data not shown).

The aim of our study was to develop DNA aptamers against *A. phagocytophilum*, or molecules expressed during the infection. Aptamers would be quintessential tools for *A. phagocytophilum* research as they could facilitate the identification of molecules involved in *A. phagocytophilum*-host interactions or the capture of the bacterium for downstream applications (culture of new strains, bacteria-enrichment for genomic studies or diagnosis). Therefore, a customized SELEX method was applied against an enriched suspension *A.* *phagocytophilum*, containing DC/RC forms, and bacterial and cellular molecules. After the selection, we demonstrated the ability of three aptamers to bind specifically to infected cells, with high affinity.

## Results

### Preparation of an enriched suspension of *A. phagocytophilum* NY18 strain

The first aim was to purify *A. phagocytophilum* NY18 strain from HL-60 cells. Three cell lysis methods were compared, allowing the recovery of both DC and RC forms of *A. phagocytophilum*. After purification of infected HL-60 cells with syringe lysis, the bacteria were recovered, with large number of cellular debris (Supplementary Fig. S1a). Contamination with grit was observed after purification by rock tumbler grit method (Supplementary Fig. S1b). These two purification methods were therefore not optimal. In contrast, purification of heavily infected HL-60 cells with the Dounce homogenizer resulted in important recovery of *A. phagocytophilum* organisms, with few host cell debris (Supplementary Fig. S1c). The viability of purified *A. phagocytophilum* organisms was confirmed after reinfection of HL-60 cells, as three days post-infection, more than 80% of cells were infected (Supplementary Fig. S1d). With this purification method, we obtained an enriched suspension of *A. phagocytophilum*, further used for aptamer selection.

### SELEX against enriched suspension of *A. phagocytophilum* and its monitoring by qPCR

SELEX was performed against the enriched suspension of *A. phagocytophilum* NY18 strain. To eliminate sequences specific to host-cell components, negative selection was performed against a lysate of non-infected host cells (HL-60).

For the first selection rounds (R01-R05), the conditions were chosen according to the recommendations found in the literature^27^. At the beginning, the selection pressure was high: competitors and negative selection were added from R03 and R04 respectively, and the number of washes was increased from R05. The evolution of SELEX was monitored by qPCR. After R06, the selection conditions were adapted, according to the results of the amplification and melting curves obtained by qPCR (Table 1)^28^. For R02 to R05, the amplification curves showed a drop in fluorescence, indicating that the ssDNA pool contained many random and non-specific sequences (Supplementary Fig. S2a). From R06, the fluorescence reached a plateau, indicating a decrease of sequence diversity^28^. For melting curves, the DNA pools at the end of the elongation phase were denatured slowly, forming stable (homoduplexes) or unstable (heteroduplexes) products. A single peak at a Tm of 64 °C was observed up to R06, corresponding to heteroduplexes, as the diversity of sequences was high and therefore led to non-specific mismatches (Supplementary Fig. S2b). From R06, we observed an increase in stable compounds (homoduplexes with a Tm of 79 °C), combined with a decrease in heteroduplexes. These results confirmed the decrease of sequence diversity, associated with the emergence of potential enriched sequences, from R06. After R07, the conditions of selection were not changed to avoid losing potential enriched sequences. SELEX was stopped after R12 since qPCR monitoring showed no difference between R11 and R12, indicating a halt in the evolution of sequences.

**Table 1:** SELEX conditions performed against the enriched suspension of *A. phagocytophilum* NY18 strain.

	ssDNA concentrations		Total number of cells		Incubation parameters			Melting curves		Adaptation of conditions
SELEX round	Quantity	Concentration	HL-60 NY18	HL-60	Temperature incubation time	Washing	Competitors	Hetero duplexes	Homo duplexes	Comments
R01	2 nmol	2 µM	3.10^7^	–	23 °C 45 min 650 rpm	1X 1 mL, 6 min	–	Presence	Absence	-
R02	0.03 nmol*	0.5 µM	3.10^7^	–	23 °C 45 min 650 rpm	1X 1 mL, 6 min	–	Presence	Absence	Decrease in the initial amount of ssDNA
R03	0.1 nmol*	0.5 µM	3.10^7^	–	23 °C 45 min 650 rpm	1X1 mL, 10 min*	BSA, tRNA, salmon sperm DNA*	Presence	Absence	Introduction of competitors
R04	0.01 nmol*	0.5 µM	3.10^7^	3.10^7^*	23 °C 45 min 650 rpm	1X 1 mL, 10 min	BSA, tRNA, salmon sperm DNA	Presence	Absence	Introduction of negative selection
R05	0.01 nmol	0.34 µM*	3.10^7^	4.10^7^*	23 °C 45 min 650 rpm	3X 1 mL, 30 min*	BSA, tRNA, salmon sperm DNA	Presence	Absence	Increased number of healthy cells and washing steps
R06	0.01 nmol	0.34 µM	2.10^7^*	4.10^7^	23 °C 30 min 650 rpm (1 h for negative selection) *	3X 1 mL, 30 min	BSA, tRNA, salmon sperm DNA	Presence	Absence	Decreased number of infected cells
R07	0.01 nmol	0.2 µM*	2.10^7^	4.10^7^	23 °C 30 min 650 rpm (1 h for negative selection)	3X 1 mL, 30 min	BSA, tRNA, salmon sperm DNA	Presence	Absence	Decrease in ssDNA concentrations
R08	0.01 nmol	0.2 µM	2.10^7^	4.10^7^	23 °C 30 min 650 rpm (1 h for negative selection)	3X 1 mL, 30 min	BSA, tRNA, salmon sperm DNA	Absence	Presence	No modifications
R09	0.01 nmol	0.2 µM	2.10^7^	4.10^7^	23 °C 30 min 650 rpm (1 h for negative selection)	3X 1 mL, 30 min	BSA, tRNA, salmon sperm DNA	Absence	Presence	No modifications
R10	0.01 nmol	0.2 µM	2.10^7^	4.10^7^	23 °C 30 min 650 rpm (1 h for negative selection)	3X 1 mL, 30 min	BSA, tRNA, salmon sperm DNA	Absence	Presence	No modifications
R11	0.01 nmol	0.2 µM	2.10^7^	4.10^7^	23 °C 30 min 650 rpm (1 h for negative selection)	3X 1 mL, 30 min	BSA, tRNA, salmon sperm DNA	Absence	Presence	No modifications
R12	0.01 nmol	0.2 µM	2.10^7^	4.10^7^	23 °C 30 min 650 rpm (1 h for negative selection)	3X 1 mL, 30 min	BSA, tRNA, salmon sperm DNA	Absence	Presence	No modifications

The stars correspond to conditions that have been modified between two successive rounds.

### Identification of aptamer candidates by NGS

**Figure 1:**
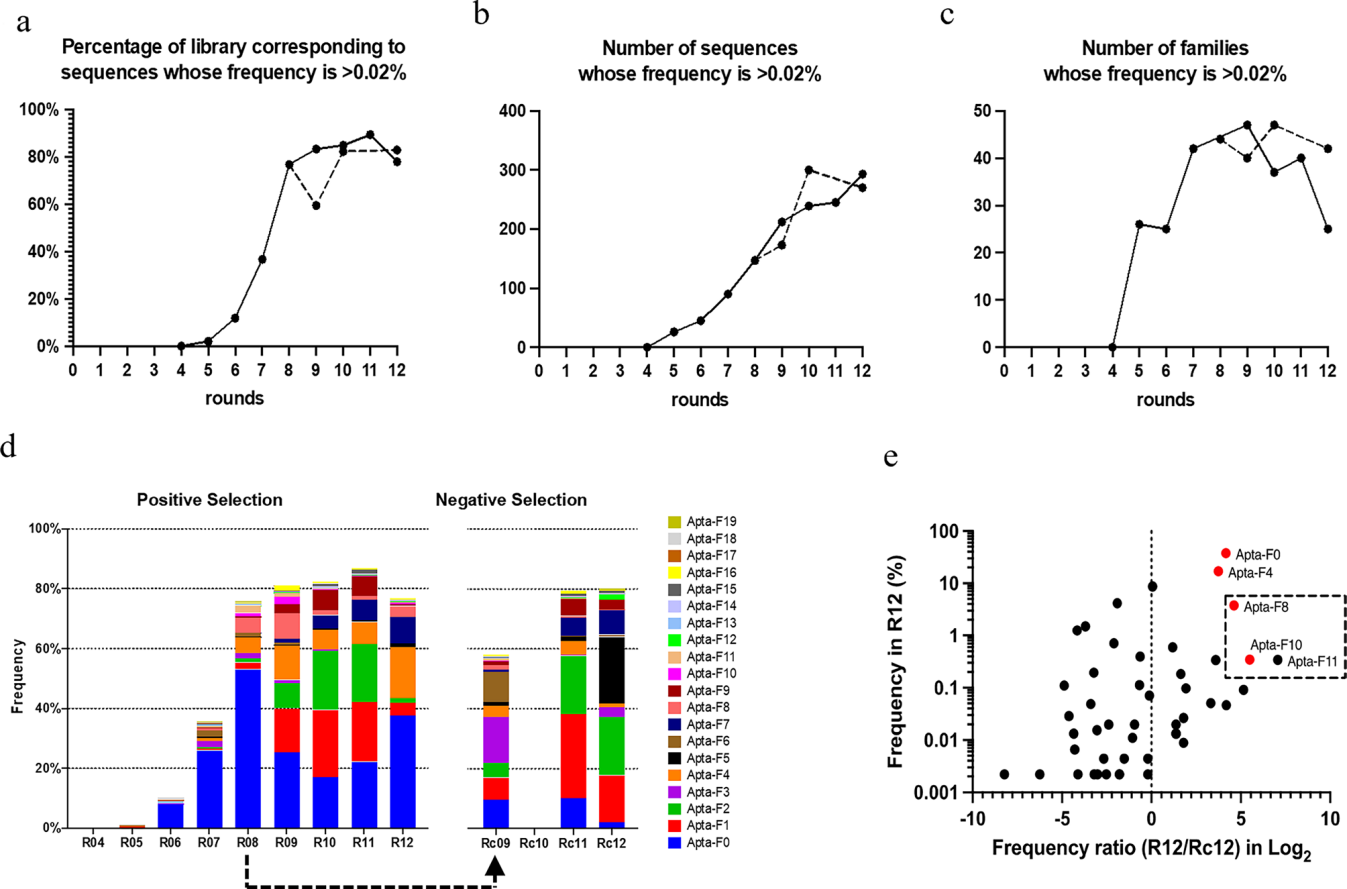
Next-generating sequencing (NGS) analysis of SELEX. Sequences whose frequency is higher than 0.02% at least in one cycle have been recovered. The library was split after the eighth round to compare the evolution of the families during four additional rounds of SELEX (positive selection, solid lines) *vs* four rounds of SELEX on cell extracts from uninfected cells (negative selection, dashed lines). (**a**) Percentage in the library corresponding to sequences whose frequency is higher than 0.02% at least in one round. (**b**) Number of sequences whose frequency is higher than 0.02%. (**c**) Number of families (sequences have been clustered in families based on an edit distance of 8). (**d**) Evolution of the frequency of the 20 most amplified families in the library during different rounds of SELEX. (**e**) Frequency of the most enriched families in the last round of SELEX (R12) compared to their frequency ratio (R12/Rc12) in Log_2_ to highlight those that are more enriched under positive selection compared to negative selection (corresponding to a ratio greater than 0). The dashed region indicates 3 families with a conserved motif. The red dots represent the candidate aptamers selected for binding evaluation.

**Figure 2:**
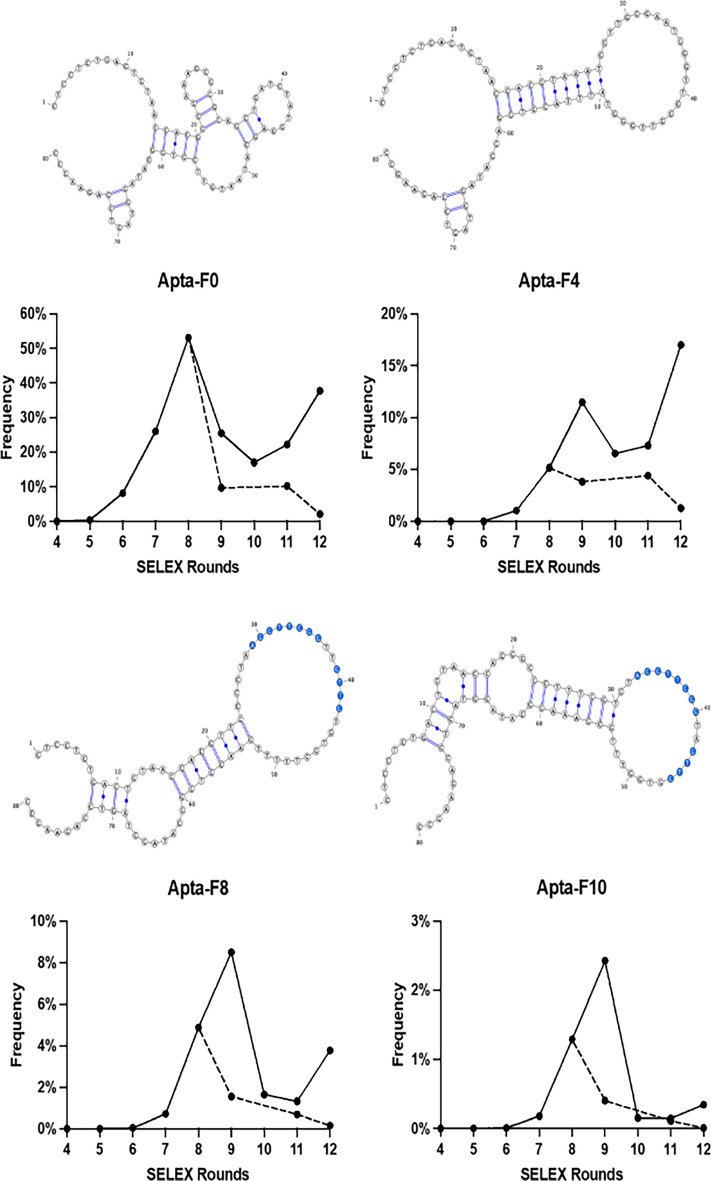
Evolution of the frequency and predicted secondary structures of the four aptamer candidates chosen for binding evaluation. The structure prediction was performed using Mfold. A conserved sequence between Apta-F8 and Apta-F10 is colored in blue. The evolution of the frequency of these aptamer candidates in rounds of positive and negative selection are presented in solid line and dashed line, respectively.

The final ssDNA pools obtained from R04 and R12 were analyzed by NGS. Over 95% of the sequences obtained by NGS consisted of 40 nucleotides, meaning that non-specific amplification was limited during the selection, partly due to the optimization of the number of PCR cycles at each round (data not shown). To focus our analysis on the most enriched sequences, we have retained for analysis only 778 sequences with a frequency greater than 0.02% in at least one cycle. As expected, the frequency of a few sequences strongly increased during SELEX (Fig. 1a,b). Among these sequences, several were grouped in a common family when they were separated by an edit distance lower than 8 (Supplementary Table S1). This clustering created 98 families. Interestingly, the number of sequences increased continuously whereas the number of families decreased after R09 (Fig. 1b,c). This was due to the fact that some sequences increase in the library while others, less adapted, decreased (Supplementary Tables S1 and S2). The presence of a conserved motif between some of the most amplified sequences was revealed by multiple alignment (Supplementary Table S3). The choice of candidate aptamers was based on the comparison of the NGS results obtained by our SELEX (R02-R12) *vs* the control SELEX, performed on HL-60 lysates (Rc09-Rc12). This original strategy allowed us to eliminate several families which, although highly enriched, were more frequent in the control SELEX. This was the case for families 1 and 2, which were progressively enriched, reaching a frequency of 4.16% and 1.50% respectively in R12, compared to 15.72% and 19.37% in the Rc12 (Fig. 1d,e and Supplementary Table S2). As these results strongly suggested that these families would have recognized molecules present in both infected and non-infected cell lysates, they were not selected as candidates for further studies. Applying this strategy, four families were chosen: family 0 (37.73% in R12 *vs *2.12% in Rc12), family 4 (17% in R12 *vs* 1.27% in Rc12), family 8 (3.78% in R12 *vs* 0.15% in Rc12) and family 10 (0.35% in R12% *vs *0.01% in Rc12) (Fig. 2).

We chose the most frequent variant of each family, named Apta-F0, Apta-F4, Apta-F8 and Apta-F10. A common structure loop for Apta-F8 and Apta-F10 was observed (Fig. 2). The primary structures of these sequences are presented in Table 2, as well as the scramble chosen for binding analysis.

**Table 2:** Nucleotide sequences of aptamers identified by NGS sequencing. The nucleotides in bold correspond to the variable region.

**Scramble**	CTCCTCTGACTGTAACCACG**AGCCGACTCGGATCTTGGTACGTGCCACTGTTGCATCGTG**GCATAGGTAGTCCAGAAGCC
Apta-F0	CTCCTCTGACTGTAACCACG**GCGAAAGCGCGCAGCTGATCTATGGAGCATAATGTTCGTG**GCATAGGTAGTCCAGAAGCC
Apta-F4	CTCCTCTGACTGTAACCACG**TAAATGGTTGGGAATGGGTTGGGTTGGGTATTTACGTGGA**GCATAGGTAGTCCAGAAGCC
Apta-F8	CTCCTCTGACTGTAACCACG**TTCCCCTAAGGTTCGGTTGTTGTGTGGTTTTTGAACGTGG**GCATAGGTAGTCCAGAAGCC
Apta-F10	CTCCTCTGACTGTAACCACG**GCCCTTTCGTGTAGGTTCGGTAGTTGGTGGTTTGCGAAAG**GCATAGGTAGTCCAGAAGCC

### Determination of binding affinities by flow cytometry analysis

**Figure 3:**
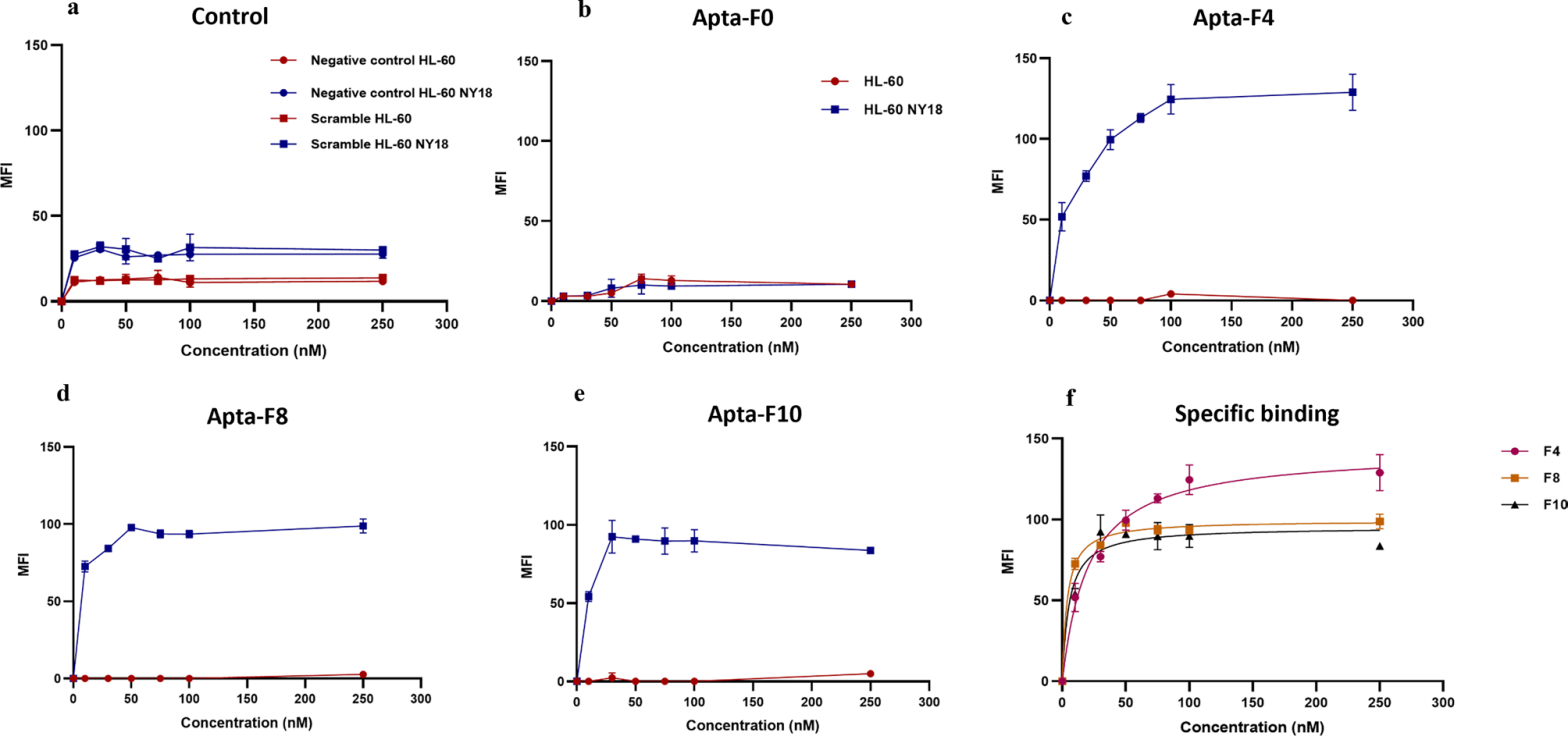
Binding study of selected sequences against HL-60 or HL-60 NY18 cells, by flow cytometry, for one experiment. Different concentrations of the conjugated aptamers (from 0 to 250 nM) were incubated with unfixed cells. (**a**) Median fluorescence intensity (MFI) values obtained for scramble or streptavidin-AF 647 nm incubated with HL-60 (red line) or HL-60 NY18 (blue line) cells. Each aptamer concentration was tested in duplicate. Error bars represent the standard deviation of the duplicates. (**b**) Result of specific binding of the aptamer against HL-60 cells (red line) or HL-60 NY18 cells (blue line). The MFI values of the scramble were subtracted from each MFI value. Each aptamer concentration was tested in duplicate. Error bars represent the standard deviation of the duplicates. (**c**) Representation of the specific binding of aptamers against HL-60 NY18 cells. The MFI values of the scramble were subtracted from each MFI value. Each aptamer concentration was tested in duplicate. Error bars represent the standard deviation of the duplicates.

The binding affinities of the selected aptamers against unfixed HL-60 cells or *A. phagocytophilum* infected cells (HL-60 NY18) were assessed by flow cytometry (Fig. 3). Aptamers were conjugated with fluorescent streptavidin (AF-647 nm). *A. phagocytophilum* were purified from the cells, labelled with CMFDA and then used to infect HL-60 cells. Different concentrations of aptamers were tested against HL-60 or HL-60 NY18 cells.

The scramble did not bind to HL-60 or HL-60 NY18, as indicated by the median fluorescence intensities (MFI) close to the negative control (streptavidin AF-647 nm only) (Fig. 3a). The absence of binding for the scramble sequence validated our cell staining parameters, as we did not observe non-specific interactions. For uninfected HL-60 cells, all aptamers obtained MFI values close to the scramble sequence, indicating that aptamers did not bind specifically to HL-60 cells.

In contrast, data obtained for Apta-F4, Apta-F8 and Apta-F10 against infected cells showed a typical saturation curve, these aptamers interacted with the infected cells in a concentration-dependent manner (Fig. 3c,d,e). The binding of these sequences was significantly higher compared to the scramble. Conversely, for Apta-F0, little or no fluorescence signal was obtained with HL-60 NY18 (Fig. 3b). Therefore, Kd of this sequence was not determined. The average of Kd and range of Bmax values obtained for three experiments were calculated from specific binding curve against HL-60 NY18 and are presented in Table 3. The average Kd was in the nanomolar range from 4.74 to 40.48 nM, depending on the sequences. The binding study by flow cytometry demonstrated the high affinities of Apta-F4, Apta-F8 and Apta-F10 against cells infected with *A. phagocytophilum* NY18 strain.

**Table 3:** Average Kd and range of Bmax values + /- standard deviation of three experiments, calculated from graphs representing sequence-specific binding.

Binding affinity	Apta-F4	Apta-F8	Apta-F10
Kd (nM)	40.48 +/− 13.2	4.74 +/− 2.85	7.55 +/− 3.02
Bmax	[142.6–199.4]	[87.04–104.1]	[95.03–107.8]

### Study of aptamer binding in HL-60 and HL-60 NY18 cells by fluorescence microscopy

The binding of aptamers was then studied by confocal microscopy, to confirm the previous results and to visualize their localization in the cells. For each experiment, aptamers were conjugated with fluorescent streptavidin (AF-647 nm), *A. phagocytophilum* was labelled with CMFDA and nuclei were labelled with DAPI.

First, aptamers were tested against a lysate of uninfected cells (HL-60) or infected cells (HL-60 NY18) to confirm that aptamers did not recognize an intracellular molecule present in uninfected cells. The results confirmed the absence of recognition of the HL-60 cell lysate, by any of the preselected aptamers and the presence of binding against HL-60 NY18 cell lysates for Apta-F4, Apta-F8 and Apta-F10 (Supplementary Fig. S3). For Apta-F0, we obtained the same binding as for the scramble sequence. This experiment confirmed that Apta-F4, Apta-F8 and Apta-F10 recognized a target only expressed in infected cells.

**Figure 4:**
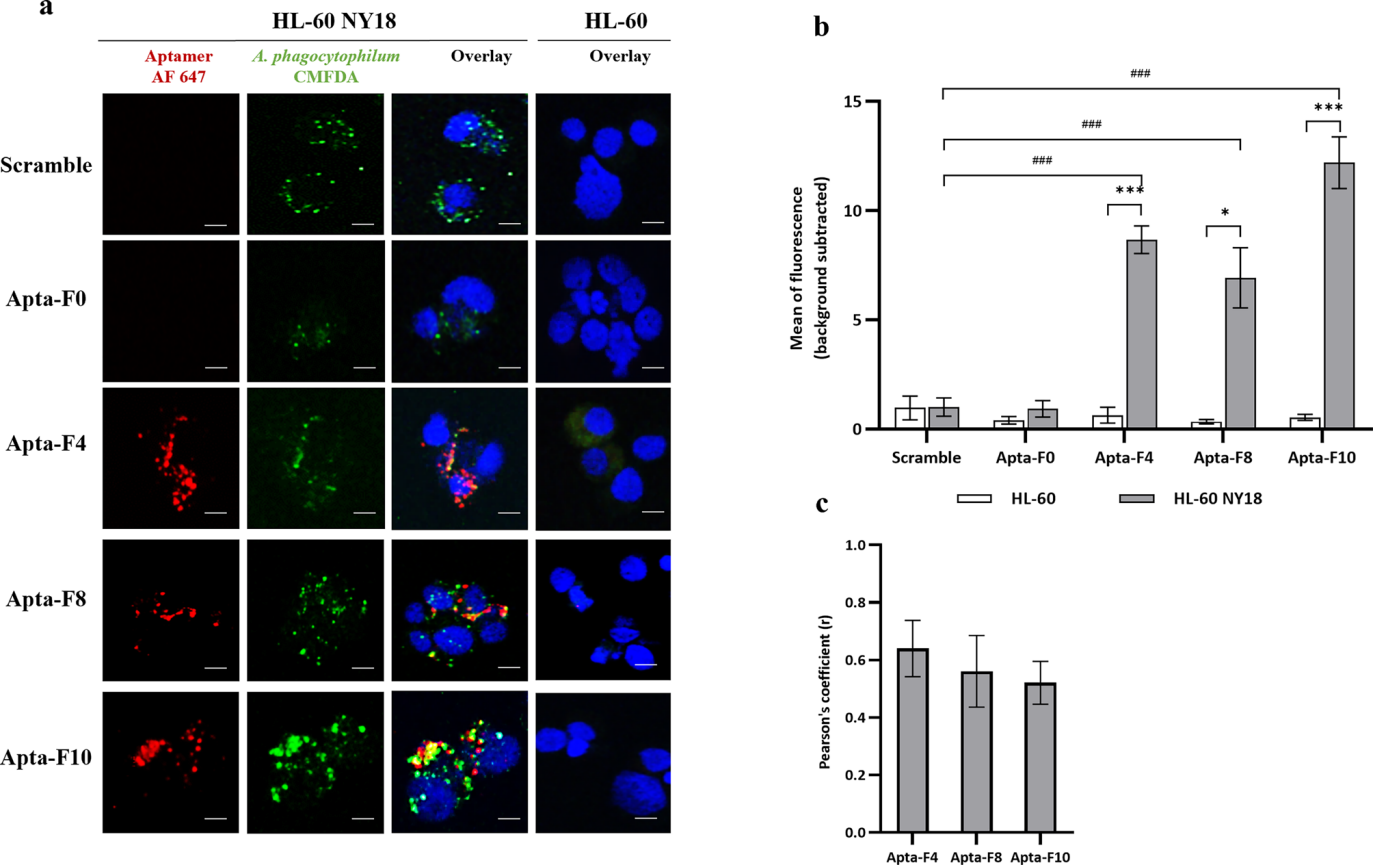
(**a**) Confocal microscopy binding studies against HL-60 and HL-60 NY18 cells. Aptamers were incubated with the unfixed cells. Nuclei were labelled with DAPI (blue) and biotinylated aptamers were pre-conjugated with streptavidin-AF-647 nm (red). *A. phagocytophilum* NY18 strain was labelled with CMFDA (green), to obtain fluorescent HL-60 NY18. Scale bars represents 5 µm. (**b**) Representation of the average fluorescence intensity of each sequence (red channel), quantified by ImageJ software. Values were obtained after subtraction of background noise. Experiments were performed in triplicates (90 cells analyzed per sequence) and error bars represent the standard deviation. A one-way ANOVA test was performed to compare the average fluorescence intensity between the different conditions. Asterisks represent significant differences (###: p < 0.0001 compared with the scramble, *: p < 0.01, ***: p < 0.0001 compared between HL-60 and HL-60 NY18 cells). **(c)** Quantitative study of colocalization by measuring the Pearson correlation coefficient, obtained for Apta-F4, Apta-F8 and Apta-F10 (JacoP PlugIn). The experiments were performed in triplicates (30 cells per experiment).

Second, the conjugated aptamers were incubated with unfixed HL-60 cells or HL-60 NY18 cells, after three days of infection. The objective was to localize the binding sites. No binding was observed for the scramble, demonstrating the absence of non-specific signal (Fig. 4a). As expected, uninfected cells were not recognized by any of aptamers. For Apta-F0, no fluorescence was observed for HL-60 NY18 cells, consistent with the low values obtained by flow cytometry. In contrast, significant binding was observed for Apta-F4, Apta-F8 and Apta-F10 in HL-60 NY18 cells, compared to the scramble (Fig. 4b). These aptamers provided an intracellular signal and recognized infected cells. Noticeably, the red and green pixels seem to overlap for Apta-F4, Apta-F8 and Apta-F10. The colocalization between aptamers (red) and *A. phagocytophilum* (green) was further analyzed, by estimating the Pearson correlation coefficient (Fig. 4c). This coefficient ranged between 0.53 and 0.63, indicating partial colocalization between the two channels. A perfect colocalization corresponds to a Pearson correlation coefficient of 1, a negative colocalization to −1 and the absence of colocalization to zero^29^. For the intermediate values between −0.5 to 0.5, no conclusion can be drawn.

**Figure 5:**
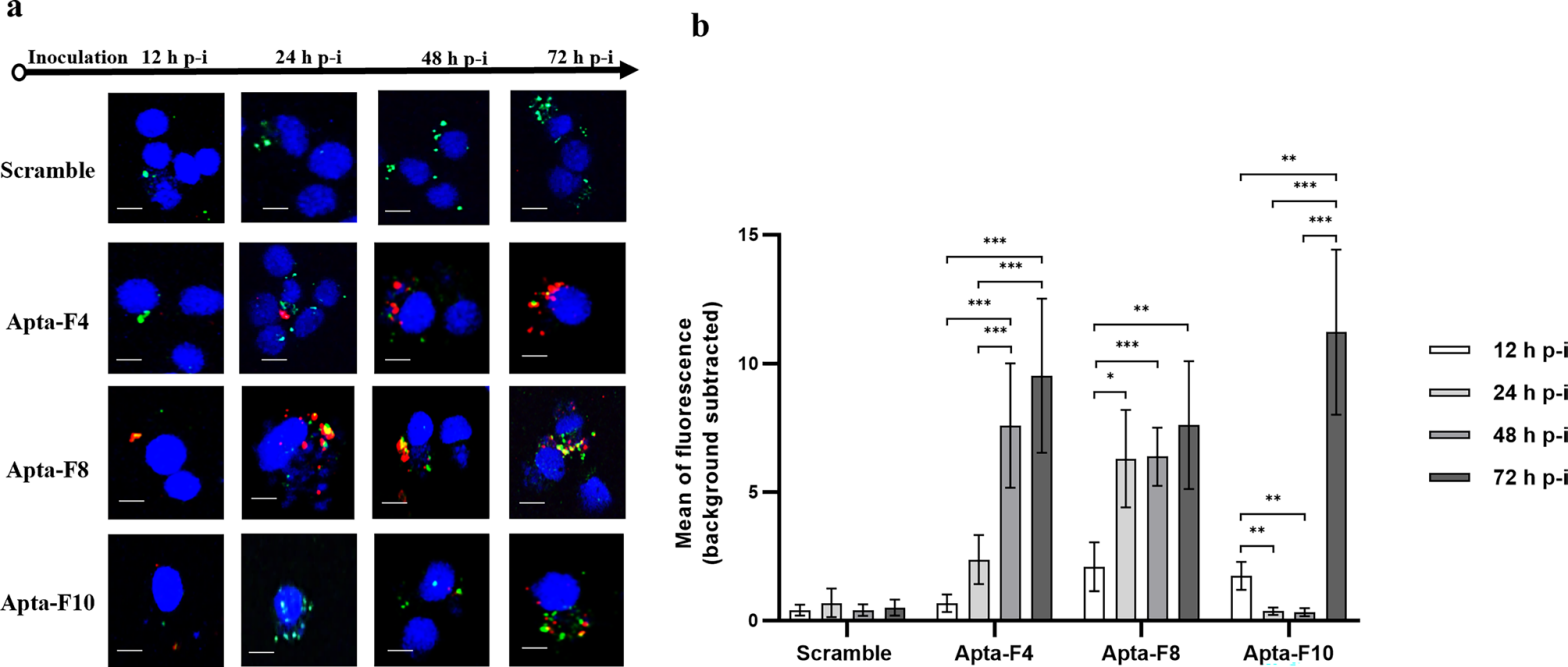
(**a**) Confocal microscopy binding studies during *A. phagocytophilum* infection. Aptamers were incubated with unfixed HL-60 NY18 cells after 12 h, 24 h, 48 h or 72 h infection. Nuclei were labelled with DAPI (blue), and biotinylated aptamers were pre-conjugated with streptavidin-AF-647 nm (red). *A. phagocytophilum* NY18 strain was labelled with CMFDA (green). Scale bars represents 10 µm. The experiments were performed in triplicates, independently of each other. (**b**) Representation of the average fluorescence intensity of each sequence (red channel), quantified by ImageJ software. Values were obtained after subtraction of background noise. Experiments were performed in triplicates (90 cells analyzed per sequence) and error bars represent the standard deviation. A one-way ANOVA test was performed to compare the average fluorescence intensity between the different time of infection. Asterisks represent significant differences (*: p < 0.01, **: p < 0.001 and ***: p < 0.0001).

Third, aptamers were incubated with unfixed HL-60 NY18 cells after 12 h, 24 h, 48 h or 72 h of infection (Fig. 5). Each sequence showed different binding profiles during infection and appeared to recognize different types of targets. Apta-F4 was able to recognize its target from 24 h of infection, with a weak binding (Fig. 5a,b). The level of binding increased between 24 h and 72 h post-infection (Fig. 5a,b). Apta-F8 binding was already observed from 12 h post-infection and increased as the infection progressed (Fig. 5a,b). Interestingly, Apta-F10 was able to bind to its target at 12 h post-infection and then only after 72 h of infection (Fig. 5a,b).

An additional experiment was performed to determine whether aptamers could be internalized by active pathways, including endocytosis. The conjugated aptamers were therefore incubated at 4 °C with HL-60 NY18 cells, harvested after three days of infection. At this temperature, no active transport occurs^30^. As expected, no binding was detected for scramble (Supplementary Fig. S4). Conversely, strong binding was observed for Apta-F4, Apta-F8 and Apta-F10. This temperature did not affect the intracellular binding of these aptamers. Endocytosis pathways do not appear to be involved in the internalization of aptamers. These results argue in favor of passive entry of aptamers into cells naturally permeabilized by *A. phagocytophilum* infection, rather than active internalization.

### Impact of trypsin pre-treatment on aptamer binding against HL-60 NY18 cells

To determine whether these aptamers recognize proteins, highly infected HL-60 NY18 cells were treated with trypsin for 10 min or 30 min, prior to aptamer incubation. Trypsin, as a serine protease, hydrolyzes proteins into small peptides.

**Figure 6:**
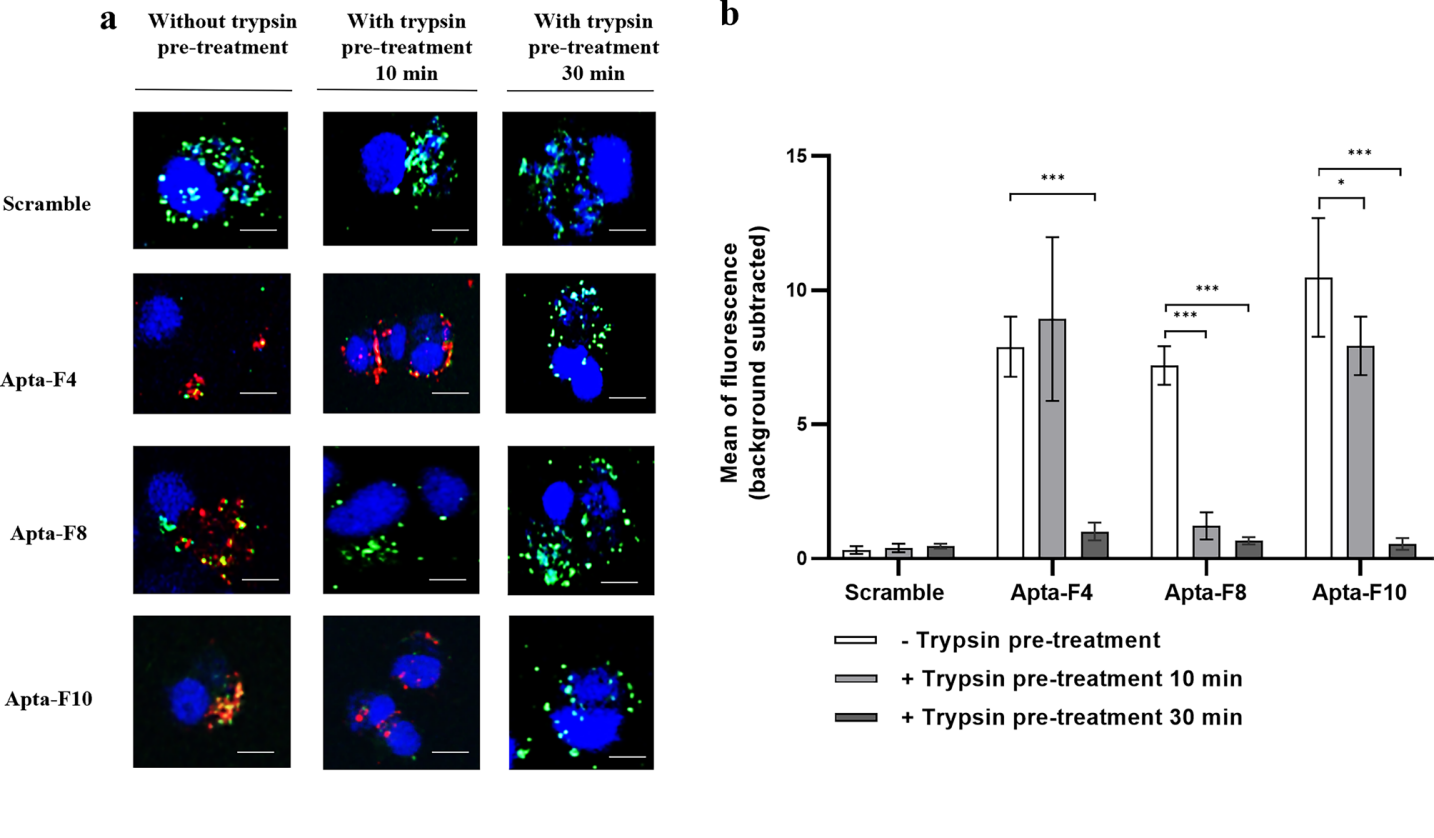
(**a**) Confocal microscopy binding studies against HL-60 NY18 cells with/without trypsin pre-treatment. After three days of infection, HL-60 NY18 cells were pre-treated with trypsin for 10 or 30 min. Aptamers were then incubated with the unfixed cells. Nuclei were labelled with DAPI (blue), and biotinylated aptamers were pre-conjugated with streptavidin-AF-647 nm (red). *A. phagocytophilum* NY18 strain was labelled with CMFDA (green). Scale bars represents 10 µm. The experiments were performed in triplicates, independently of each other. (**b**) Representation of the average fluorescence intensity of each sequence (red channel), quantified by ImageJ software. Values were obtained after subtraction of background noise. Experiments were performed in triplicates (90 cells analyzed per sequence) and error bars represent the standard deviation. A one-way ANOVA test was performed to compare the average fluorescence intensity with the control (without trypsin). Asterisks represent significant differences (*: p < 0.01, **: p < 0.001 and ***: p < 0.0001).

The impact of trypsin pre-treatment on aptamer binding is presented in Fig. 6. For Apta-F8, binding was considerably reduced after 10 min of trypsin treatment (Fig. 6a,b**)**. For Apta-F4 and Apta-F10, a reduction of binding was observed after 30 min of treatment (Fig. 6a,b). However, since trypsin was incubated with the infected cells for 30 min, we could therefore question the reality of its complete inactivation. Trypsin may have degraded the fluorescent streptavidin coupled to aptamers. The same experiment was therefore performed using aptamers directly coupled to AF-647 nm. No binding was observed for all the sequences after 30 min of trypsin pre-digestion (Supplementary Fig. S5). This experiment confirm that trypsin was well inactivated. The absence of binding indicates that the aptamer targets have been digested by trypsin. These aptamers seem to recognize protein structures.

### Study of aptamer binding in tick cells by fluorescence microscopy

Aptamer binding was tested against ISE6 cells infected by *A. phagocytophilum* NY18 strain or by the ovine strain NV2Os. The results were observed by confocal microscopy and compared with those obtained by binding against non-infected ISE6 cells. Aptamers were conjugated with fluorescent streptavidin (AF-647 nm) and nuclei were labelled with DAPI. Unfortunately, the kinetics of *A. phagocytophilum* infection in tick cells did not allow us to label *A. phagocytophilum* in this model of infection. CMFDA-labelled *A. phagocytophilum* remain fluorescent for up to 4 days^31^. However, approximately two weeks are required to observe infection with *A. phagocytophilum* in ISE6 tick cells. A previous assay using CMFDA was performed in tick cells and failed to label *A. phagocytophilum* (data not shown).

**Figure 7:**
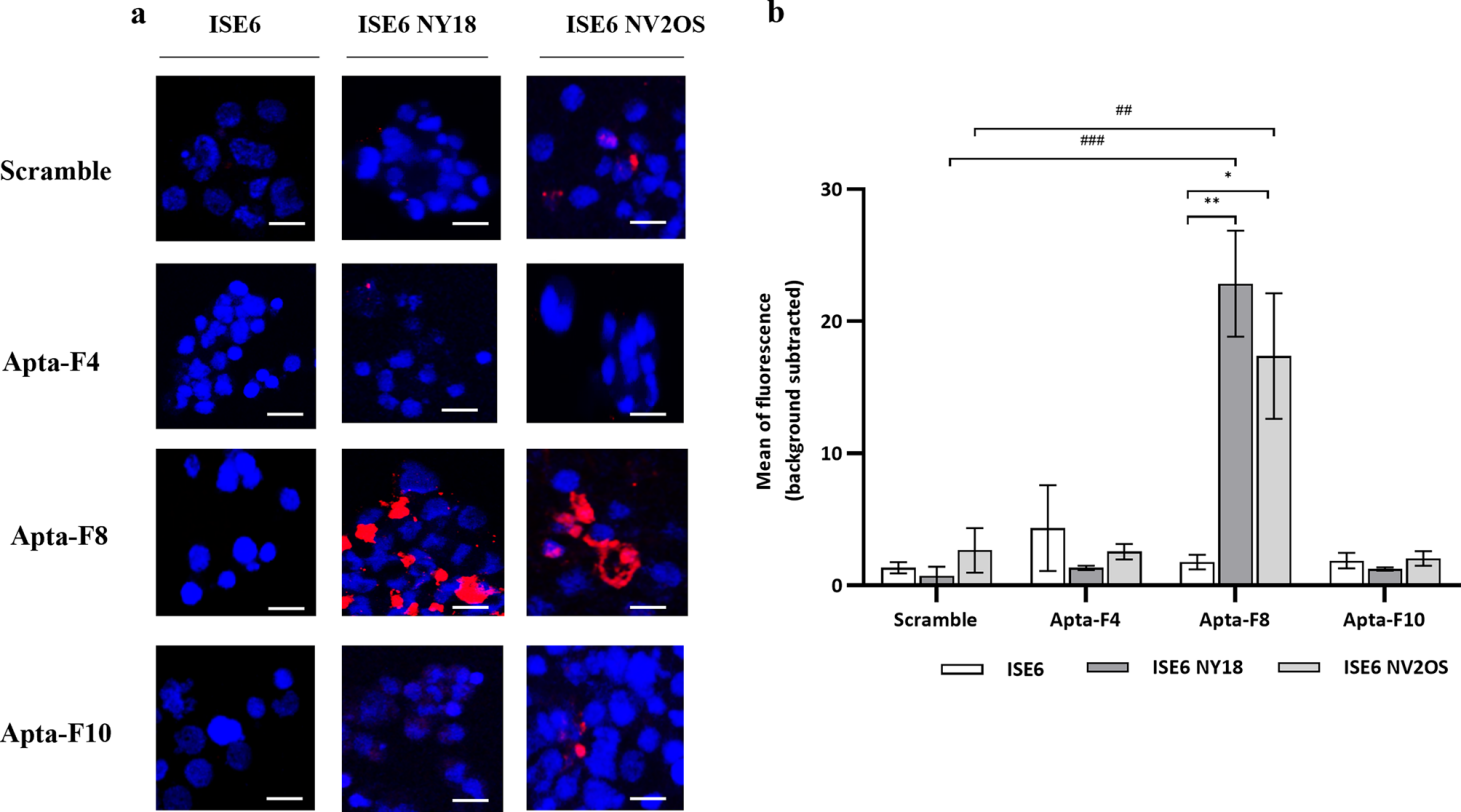
(**a**) Confocal microscopy binding studies against ISE6 cells, ISE6 NY18 cells and ISE6 NV2Os cells. Aptamers were incubated with the unfixed cells. Nuclei were labelled with DAPI (blue), and the biotinylated aptamers were pre-conjugated with streptavidin-AF-647 nm (red). (**b**) Representation of the average fluorescence intensity of each sequence (red channel), quantified by ImageJ software. The values were obtained after subtraction of background noise. Experiments were performed in triplicates (90 cells analyzed per sequence) and error bars represent the standard deviation. A one-way ANOVA test was performed to compare the average fluorescence intensity between the different conditions. Asterisks represent significant differences (##: p < 0.001, ###: p < 0.0001 compared with the scramble, *: p < 0.01 and **: p < 0.001 compared between ISE6, ISE6 NY18 and ISE6 NV2Os cells).

For non-infected ISE6 cells, no binding was detected for all aptamers (Fig. 7). When these cells were infected with the NY18 strain, only Apta-F8 was able to bind infected ISE6 cells (Fig. 7a,b).

For ISE6 cells infected with the ovine strain NV2Os, low non-specific interactions were observed for the scramble (Fig. 7a,b). For Apta-F4 and Apta-F10, the binding was not statistically different from that of the scramble. In contrast, Apta-F8 demonstrated significant binding. This aptamer therefore recognized a molecule also expressed during the invertebrate infection model. However, we cannot draw any conclusions about the localization of its binding site.

## Discussion

Developing aptamers as new tools is a real opportunity for the study of *A. phagocytophilum*, both for basic research and for downstream applications. To reach this goal, DNA aptamers were chosen as they are more advantageous over RNA aptamers, due to their higher stability, easier selection and amplification processes, longer shelf-life and low cost^32^. As a target, we chose to work with the NY18 strain of *A. phagocytophilum*, isolated from a human patient in the USA^14^. This strain is interesting for studying the interactions between the bacterium and its invertebrate/vertebrate host since it can be grown rapidly in both culture models^33^.

Selecting aptamers against strict intracellular pathogens is challenging and has been described only once in the literature to date^26^. In our study, we implemented a customized selection method with original strategies that could help researchers in the selection of aptamers against strict intracellular targets, both pathogens and molecules expressed during cell infection. The main challenge was to obtain aptamers specific to molecules related to *A. phagocytophilum* infection, and not aptamers directed against molecules from uninfected cells as the presence of cell debris cannot be avoided^34^. Even if the purification of *A. phagocytophilum* by a Dounce homogenizer provides a suspension enriched in bacteria, a small amount of host cell components is still present. To counteract this problem, we applied negative selection against host cell lysates at each round starting at R04. In addition, the selection conditions were monitored according to the results of qPCR, as proposed in a previous study^28^and to NGS. The results obtained with these two methods were consistent with each other. We therefore strongly advise researchers to apply qPCR analysis at each round of selection and to adapt their SELEX conditions accordingly. NGS is a more expensive method and can therefore be applied at the end of the selection process to confirm qPCR results and identify aptamer sequences. According to the literature, the enrichment against pure targets is usually observed between R08 and R12 and depends on the conditions of selection^34^. A previous study has selected aptamers against *Enterococcus faecalis* with the enrichment observed from R09^35^. Negative selection was not applied for each round and only two competitors were used (BSA and salmon sperm DNA). Interestingly, we succeeded in a rapid sequence enrichment from R06, despite the complexity of our target. We applied more stringent selection conditions than those found in the literature, with a negative selection performed in each round, the use of three competitors at high concentration and the large number of washing steps. However, despite the negative selection, it is not uncommon to observe the enrichment of non-specific sequences, which can lead to the SELEX failure. To identify non-specific sequences, an original strategy was applied from R09, by performing in parallel a control SELEX against host cell components. The comparison of the NGS results obtained with the control SELEX and our SELEX helped us select the best potential candidates (Apta-F0, Apta-F4, Apta-F8 and Apta-F10) for further binding studies. As expected, several sequences were more enriched in the control SELEX, despite negative selection, and were consequently eliminated.

Despite its high frequency, Apta-F0 was not able to bind to infected lysates/cells. This phenomenon is not uncommon and could result from its random presence in the selected pool^36^. In contrast, Apta-F4, Apta-F8 and Apta-F10 have interesting properties. First, they demonstrated no binding to components of uninfected cells, confirming that our selection strategy was effective. Second, these three aptamers displayed a high affinity, in the nanomolar range, against infected cells (HL-60 NY18). Third, Apta-F4, Apta-F8 and Apta-F10 were characterized by an intracellular binding, with a partial colocalization with *A. phagocytophilum*. *A. phagocytophilum* infection weakens the HL-60 cell membranes. After 72 h of infection, most of the infected HL-60 cells begin to lyse and the remaining intracellular morulae are dispersed^37^. Thus, the entry of aptamers in the unfixed infected cells could be explained through two hypotheses. Given the fragility of the cell membrane, aptamers could enter the infected cells and bind to an intracellular target, or they could recognize a receptor present at the surface of infected cells and then be internalized. Such a process of aptamer internalization via binding to a receptor membrane followed by an endocytic pathways (clathrin-mediated endocytosis, caveolin-mediated endocytosis or macropinocytosis) has been already described^38^. To test this hypothesis, endocytosis was inactivated with an incubation temperature of 4 °C^30^. Interestingly, intracellular binding of Apta-F4, Apta-F8 and Apta-F10 was not affected at this temperature. Passive entry of aptamers into cells naturally permeabilized by *A.* *phagocytophilum* infection seems more likely than active internalization by recognition of a surface target. However, these very preliminary studies do not rule out the possibility that both routes of entry could be involved. Further work is needed to investigate these hypotheses.

Finally, the target of Apta-F8 is also expressed in the invertebrate model infected with two different strains: the American human NY18 strain and the European ovine strain NV2Os. Apta-F8 seems to recognize a molecule expressed by a pathway common to both cell models during the infection, or *A. phagocytophilum* itself. In contrast, Apta-F4 and Apta-F10 only bind to the infected vertebrate model HL-60 NY18. The mechanisms of interactions in the vertebrate/invertebrate cell model remain poorly studied and little known. Some studies have shown that *A. phagocytophilum* employs common strategies in HL-60 and tick cells, such as the inhibition of apoptosis and the generation of ROS species, while others pathways are specific to the cellular model^18,39,40^. The three selected aptamers are therefore particularly interesting for a better understanding of the interactions between bacteria and cells in the vertebrate and/or invertebrate model during their infection by *A. phagocytophilum*.

At this stage of the study, we cannot conclude that aptamers recognize *A. phagocytophilum*, given the colocalization results. The results obtained after trypsin pre-treatment on highly infected cells gave us an indication as to the molecular composition of targets. Given the absence of binding after treatment, the three aptamers certainly recognize a protein structure that can be digested by proteases. This result could indicate that aptamers recognize, prior to internalization by endocytosis, a surface receptor whose structure is at least partially proteinaceous. However, we showed that aptamers recognized their target at 4 °C, a temperature at which endocytosis pathways are inactive. Based on these results, the most likely hypothesis is that our aptamers could recognize an intracellular target. A previous study showed that trypsin can be internalized by mammalian cells ^41^. Other studies have shown an alteration in cellular functions following the use of trypsin, possibly due to an intracellular action ^42,43^. In our case, given the fragility of the membrane and the long incubation time used (30 min), trypsin was probably able to degrade or reduce the expression of intracellular proteins. The selection was performed on a lysate of infected cells enriched in *A. phagocytophilum* organisms. Thus, the potential targets of these aptamers could be a protein on the surface of bacteria (DC or RC forms), or bacterial/cellular molecules secreted during infection. The kinetic studies performed in HL-60 NY18 cells demonstrated that the targets of the different aptamers are expressed at different times of infection, with probably different functions during the infection process. The target of Apta-F8 is expressed earlier from 12 h of infection. At this time of infection, the internalized DC bacteria start to become RC forms, to initiate the replication^11^. For Apta-F4, the target is expressed later, from 24 h post-infection, which corresponds to a majority of RC forms within individual inclusions in host cells^11^. Apta-F10 was only able to bind its target at two time points during the infection: at 12 h and 72 h. At both time points, *A. phagocytophilum* is mainly present in its DC form^11^.

Of importance, most of the molecules implicated in the infection by *A. phagocytophilum* are not identified and/or their functions are hypothetical^44^. For DC forms, only a few adhesins/invasins have been studied so far, such as Asp14, OmpA or AipA involved in the adhesion and entry into cells^45–47^. RC forms are known to express some specific proteins, mostly implicated in replication. For example, APH-0032 is found only on the surface of the RC forms and is located on the vacuole membrane, expressed from 20 h post-infection^48^. Some proteins are expressed commonly on the surface of both forms, such as the AmpA protein, which accumulates throughout the infection^49^. Bacterial proteins are also secreted via the type IV secretion system (T4SS)^10^. For example, the protein AnkA is secreted and crossed the vacuolar membrane to reach the nucleus of host cells, to decrease the generation of ROS species^50^. This protein is also able to bind to nuclear proteins, ATC-rich sequences, and transcriptional regions of the CYBB locus. Another T4SS protein, Ats-1, secreted into the host cell cytoplasm, is located in the mitochondria and inhibits apoptosis^51^. Ats-1 also inhibits autophagy by binding host-cell proteins located on the surface of the cytoplasmic vacuole where the RC forms develop. During infection, host cells express specific proteins, such as proteins located on the surface of cytoplasmic vacuoles (such as Beclin-1 and LC3), proteins of the endosome recycling pathway (Rab proteins) and proteins involved in plasma lipid transport (cholesterol)^10^. Finally, it has been shown that *A. phagocytophilum* infection leads to the activation of multiple host tyrosine kinases, mitogen-activated protein kinases (ERK1-2), and the production of cytokines/chemokines^10,18^. All these proteins could be potential targets for our aptamers. With regard to surface receptors, only PSGL-1 is currently identified as a key receptor for *A. phagocytophilum* entry into human neutrophils^52^. The presence of PSGL-1 on the surface of HL-60 cells is considered to be a key factor in their permissiveness towards *A. phagocytophilum*^52^. Given the lack of binding to uninfected HL-60 cells, it is unlikely that this receptor is a target for these aptamers. The next step is therefore to identify the targets of our aptamers, to determine whether they are known or unknown proteins and to explore in depth their role in cellular infection by *A. phagocytophilum*, in vertebrate and/or invertebrate models.

In conclusion, our work opens the opportunity to enhance research, diagnostic, and therapeutic approaches. Aptamers with the ability to identify cells infected by *A. phagocytophilum* may improve our understanding of the mechanisms of infection interactions. This could lead to the development of novel therapeutic strategies and potentially allow the delivery of drugs specifically targeting infected cells. The development of a method for capturing the bacterium in blood samples is a longer-term prospect, requiring further development, as target identification remains to be done. This method looks promising for the isolation and enrichment of *A. phagocytophilum* from blood samples, for diagnostic purposes and in-depth genomics studies.

## Materials and methods

### Culture of *A. phagocytophilum*

The human-derived promyelocytic leukemia cell line HL-60 was purchased from ATCC (CCL-240™, Manassas, VA, USA) and maintained in culture in IMDM medium (Thermo Fisher Scientific,), supplemented with 20% fetal calf serum (Thermo Fisher Scientific, Waltham, USA) in 75 cm^2^ flasks. The American human NY18 strain of *A. phagocytophilum* was maintained in HL-60 cells (HL-60 NY18), as previously described^14,31^. To monitor *A. phagocytophilum* infection, 100 µL of cell suspension were cytocentrifuged (Shandon Cytospin®, Thermo Fisher Scientific, Waltham, USA) and stained with Hemacolor® kit (Merck, Darmstadt, Germany) for microscopic examination. Following Hemacolor® staining, a HL-60 cell was considered microscopically infected when at least one morula was observed at 1000 × magnification. The percentage of microscopically infected cells was determined in three microscopic fields by dividing the number of infected cells by the total number of HL-60 cells. One hundred microliters of HL-60 cells infected at > 70% were added to a new flask of non-infected HL-60 cells at a final concentration of 2.10^5^ cells/mL. The cells were then incubated at 37 °C with 5% CO_2_.

ISE6 embryonic tick cells were maintained according to a previous study^53^. ISE6 tick cells were grown in 5 mL of L15B300 medium in a 25 cm^2^ flask with a pH adjusted to 7.5. The culture medium for the infected cells was supplemented with 0.1% NaHCO_3_ and 10 mM HEPES (Thermo Fisher Scientific). Infected and uninfected cells were incubated at 34 °C. The European ovine strain NV2Os of *A. phagocytophilum*^54^ (whose genome accession number is GCA_000689635.2) and the American human strain NY18 were maintained in ISE6 tick cells: 200 µL of tick cell suspension infected at > 70% were transferred to a new flask of uninfected cells. Infections were monitored as described above, by cytocentrifugation and Hemacolor® staining.

### Preparation of an enriched suspension of *A. phagocytophilum* NY18 strain

DC infectious and RC replicative forms were purified from HL-60 NY18 cells, to obtain an enriched suspension of *A. phagocytophilum*. The infected cells (2.10^7^ HL-60 NY18) were harvested by centrifugation at 500 × g for 10 min and three cell lysis methods were compared: syringe lysis, Dounce homogenizer and rock tumbler grit. For the first method, the cell pellet was recovered in 4 mL of PBS (Thermo Fisher Scientific, Waltham, USA) and then lysed via syringe passages, 5 times (5-cc tuberculin syringes, 27-G needles)^31^. For the second method, the cell pellet was harvested in 10 mL of PBS and was lysed mechanically using a Dounce homogenizer for 20 round trips. For the rock tumbler grit method, cells were suspended in 1.5 mL of IMDM medium and lysed by vortexing twice for 30 s with 60–90 grade silicon carbide^55^. Regardless of the cell lysis method, most cellular debris were removed and pelleted after centrifugation at 750 × *g* during 5 min. The supernatant was filtered with a 2 µm filter (Merck, Darmstadt, Germany) to remove the remaining debris. *A. phagocytophilum* organisms were finally pelleted by centrifugation at 10,000 × *g* for 10 min and resuspended in 100 µL of PBS. The final suspension was observed after cytocentrifugation and staining. The viability of the purified bacteria was tested by reinfecting HL-60 cells (2.10^5^ cells/mL) with *A. phagocytophilum* enriched suspension obtained by the three lysis methods. After three days of infection, 100 µL of cell suspension were observed following cytocentrifugation and staining. The lysis method that recovered a maximum of *A. phagocytophilum* with the least amount of host cell debris was chosen as the target for aptamer selection.

### SELEX library, primers, and aptamer sequences

The ssDNA library was prepared and purified by polyacrylamide gel electrophoresis (PAGE), by Eurogentec (Seraing, Belgium). It contains a region of 40 random nucleotides flanked by 20 nucleotide-long constant sequences for primer hybridization (5′-CTCCTCTGACTGTAACCACG 40N GCATAGGTAGTCCAGAAGCC-3′)^56^. A forward primer (5′-CTCCTCTGACTGTAACCACG-3′) was purchased from Eurofins Genomics (Ebersberg, Germany), and a phosphorylated reverse primer (5′-Phosphate GGCTTCTGGACTACCTATGC-3′) was synthesized and purified by high performance liquid chromatography (HPLC) at Eurogentec (Seraing, Belgium). 5’end biotinylated aptamer sequences were synthesized and purified by high purity salt free (HPSF) at Eurofins (Ebersberg, Germany). Oligonucleotides were stored at a concentration of 100 µM at − 20 °C.

### SELEX against *A. phagocytophilum* or molecules expressed during the infection

**Figure 8:**
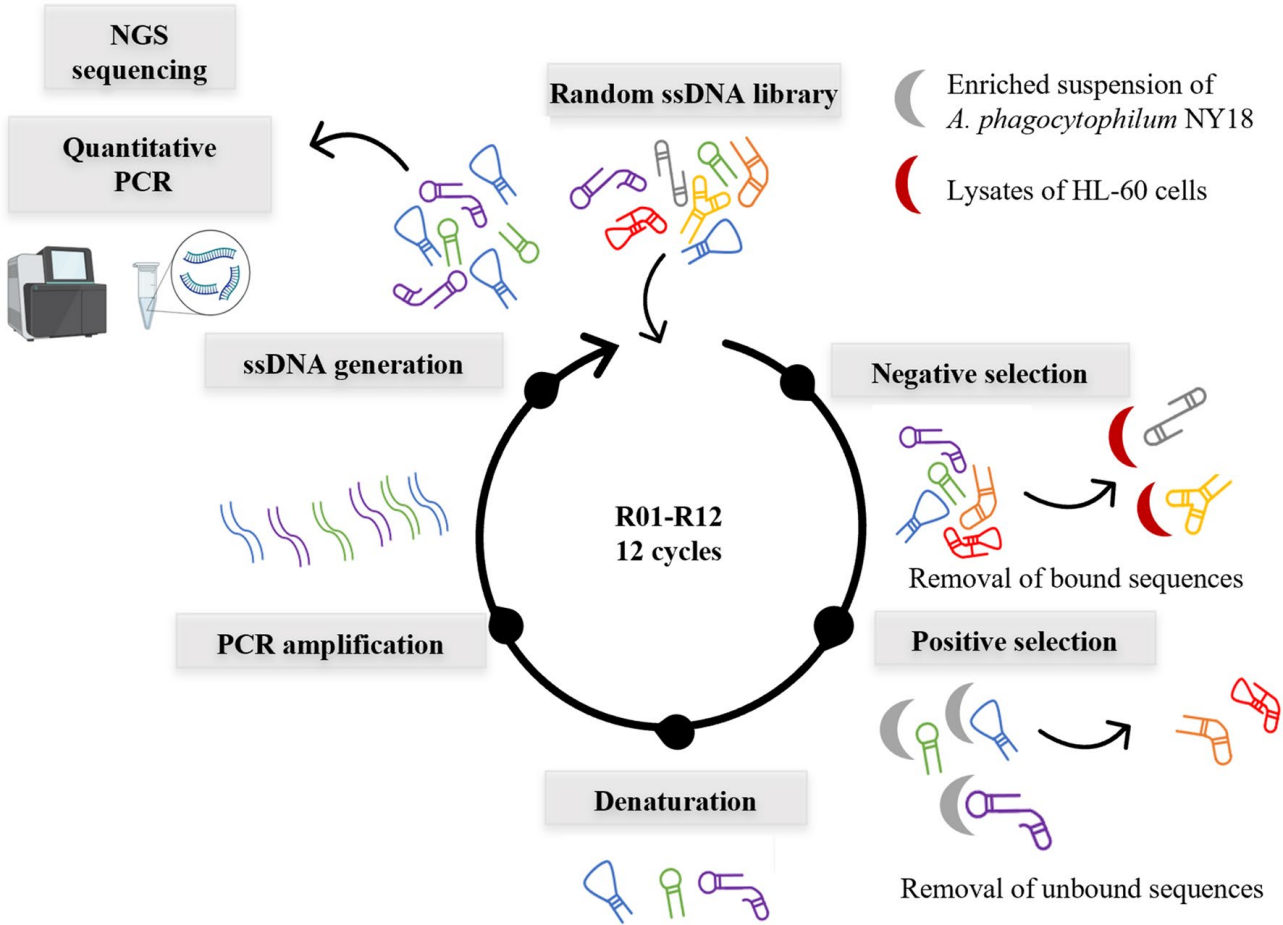
Schematic illustration of the SELEX performed against enriched suspension of *A. phagocytophilum* NY18 strain. The final ssDNA pool was analyzed by qPCR and NGS for enrichment analysis. Negative selection was introduced from R04 against HL-60 cell lysates.

SELEX was performed against the enriched suspension of *A. phagocytophilum*, according to the conditions presented in Table 1 and Fig. 8. For the first round (R01), 2 nmol of the ssDNA library were prepared in the binding buffer A, composed of PBS with CaCl_2_ and MgCl_2_ (0.9 mM CaCl_2_, 0.49 mM MgCl_2_, 2.6 mM KCl, 1.47 mM KH_2_PO_4_, 137 mM NaCl, 8.05 mM Na_2_HPO_4_, Thermo Fisher Scientific, Waltham, USA) in a final volume of 1 mL. The preparation was heated for 5 min at 95 °C, cooled on ice for 3 min to allow folding of aptamers before being incubated at 23 °C. The ssDNA library was then incubated with the enriched suspension of *A.* *phagocytophilum* at 23 °C for 45 min at 650 rpm (Eppendorf ThermoMixer 1.5) in 1.7 mL Eppendorf DNA Lowbinding (Dutscher, Issy-les-Moulineaux, France). After incubation, unbound sequences were removed after centrifugation at 10,000 × g for 6 min, followed by several washing steps of *A. phagocytophilum*-enriched pellet with 1 mL of binding buffer A. The bound sequences were eluted by heating at 95 °C for 10 min and the supernatant, containing the sequences, was recovered after centrifugation at 10,000 × g for 10 min. After phenol–chloroform DNA extraction, PCR amplification was carried out. The amplification of heterogeneous sequences can lead to the formation of non-specific PCR products, named “PCR-by-products”, due to primer-primer hybridization or binding of primers to complementary bases, present in the variable region^57^. As the amplification cycles progress, these “PCR-by-products” accumulate^57^. The optimization of the number of amplification cycles at each SELEX is therefore important to limit this phenomenon. A range of PCR cycles from 6 to 25 cycles was therefore performed at each SELEX round in a 25 µL reaction volume. The PCR mix included GoTaq Flexi Buffer 1X (Promega, Wisconsin, USA), 200 µM of dNTPs (Invitrogen, Waltham, USA), 2.5 mM of MgCl_2_, 1.25 units of GoTaq G2 Hot Start Polymerase, 2.5 µL of DNA template and 0.5 µM of forward and phosphorylated reverse primers. All PCR reactions were conducted according to the following program: an initial DNA denaturation at 95 °C for 2 min, followed by 6 to 25 cycles of denaturation (6, 8, 10, 12, 15, 20 and 25 cycles) at 95 °C for 30 s, annealing at 58 °C for 30 s, elongation at 72 °C for 15 s, finished by a final extension at 72 °C for 60 s. PCR products were revealed on a 4% agarose gel stained with BET in TAE 1X buffer at 90 V for 2 h. The optimal number of PCR cycles was then applied for large-scale PCR, performed in a final volume of 2 mL (50 µL of mix reaction per tube). All the amplification products were pooled and purified using the Nucleospin™ gel and PCR clean-up kit (Macherey–Nagel, Duren, Germany). The purity and concentration of the purified PCR product were checked and quantified by ImageJ software after gel electrophoresis with a quantitative size marker (Quick-Load Purple Low Molecular Weight DNA Ladder, NEB, Massachusetts, USA). All purified PCR products (2 µg for each reaction) were converted into ssDNA by lambda exonuclease digestion. The generation, purification and quantification of ssDNA were performed as previously described^58^.

A total of 12 cycles of SELEX (R01-R12) were performed (Table 1**, **Fig. 8). During the selection process, the conditions were modified by adding BSA (1 mg/mL, Merck, Darmstadt, Germany), tRNA (5 mg/mL Merck, Darmstadt, Germany) and salmon sperm DNA (0.1 mg/mL, Thermo Fisher Scientific, Waltham, USA) as non-specific competitors from R03, by increasing the number and time of washing steps (from one to five washing cycles, from 6 to 30 min), by decreasing the incubation time (from 45 to 30 min) and the number of infected cells (from 3.10^7^ to 2.10^7^ cells).

To avoid selection of non-specific sequences, negative selection against uninfected host cell debris, derived from HL-60 cells lysed with a Dounce homogenizer, was performed from R04 before the positive selection. The ssDNA pools were first incubated with uninfected HL-60 cell debris for 45 min at 23 °C agitated at 650 rpm. After centrifugation at 10,000 × g for 10 min, unbound sequences in the supernatant were recovered and used for positive selection. The number of HL-60 cells used for negative selection was increased as the selection cycles progressed (from 3.10^7^ to 4.10^7^ cells). After the eighth round, the library was split in half: one half was used for four more rounds (R09–R12) under the same conditions (positive selection), while the other half was used for four more rounds (named Rc09–Rc12) against host cell debris from uninfected HL-60 cells (negative control selection).

### Monitoring the evolution of SELEX by quantitative PCR

The evolution of SELEX was monitored by qPCR and melting curve analysis, performed on the final ssDNA pool obtained at each round of SELEX (R02-R12). Quantitative PCRs were conducted in a reaction volume of 20 µL, composed of 1X Luminaris Color HiGreen qPCR Master Mix (Thermo Fisher Scientific, Waltham, USA), 5 µM of aptamer primers and 2 µL of ssDNA pools, diluted at 1:100 in ultrapure water. The program consisted of an initial denaturation step at 95 °C for 10 min, followed by 40 cycles at 95 °C for 10 s, 58 °C for 15 s and 72 °C for 30 s. Melting curves were obtained at the end of qPCR by heating products to 95 °C for 15 s, cooling it down to 58 °C for 1 min and heating to 95 °C with an increment of 0.03 °C/s. The samples were amplified in duplicates with no-template controls in each run to check for contamination. The amplification curves and melting curves were analyzed with LightCycler 480 software (version 1.5, Roche, Bale, Switzerland).

### Next-generation sequencing

Aliquots of the library recovered from R04 to R12 for positive selection and from Rc09, Rc11 and Rc12 for negative selection were analyzed by Next-Generation Sequencing (NGS) on a iSeq100 instrument (Illumina), as previously described^59^. For each round, tens of thousands of sequencing reads were analyzed. Analysis was performed using a homemade software (PATTERNITY-seq), as previously described^59^. Access to this software is provided by the MIRCen aptamer platform^60^. All fastq files are available on the European Nucleotide Archive (ENA) at http://www.ebi.ac.uk/ena/data/view/PRJEB62495. First, sequences of 35 to 42 nucleotides between primer binding sites were recovered. The frequency of each sequence in each round was then calculated. All sequences with a frequency of at least 0.02% in a round were clustered into families based on a Levenstein distance of 8 (i.e., all sequences with less than 8 substitutions, deletions or insertions are grouped into the same family, Supplementary Table S1). The 0.02% threshold was chosen using Poisson's Law to tolerate a percentage error of less than 30%. The frequency of each family was then calculated for each round (Supplementary Table S2). Multiple alignment of families was performed by MultAlin (Supplementary Table S3), and structure prediction was performed using Mfold software. NGS analysis was used to select candidate aptamers for binding studies.

### Determination of binding affinity by flow cytometry analysis

The chosen aptamers and the scramble sequence were synthesized with a 5’-end biotinylation **(**Table 2**)**. The scramble corresponds to a sequence randomly designed and not selected during the SELEX. This sequence was therefore used as a control to evaluate the presence of non-specific interactions. First, the biotinylated aptamers were coupled to streptavidin-AF 647 nm (Thermo Fisher Scientific, Waltham, USA) using a 3:1 aptamer: streptavidin-AF 647 nm ratio with 1 µM of aptamer. The complex was incubated at 4 °C for 30 min in the binding buffer A. For Kd determination, different concentrations of aptamers were prepared (0 nM, 30 nM, 50 nM, 75 nM, 100 nM and 250 nM) in 100 µL of binding buffer A with 1 mg/mL of BSA, 5 mg/mL of tRNA and 0.1 mg/mL of salmon sperm DNA.

Second, *A. phagocytophilum* was labelled with Cell Tracker 5-chloromethylfluorescein diacetate (CMFDA, Thermo Fisher Scientific, Waltham, USA), to identify *A. phagocytophilum* infected cells^31^. The bacteria were purified from HL-60 NY18 cells using a Dounce homogenizer and filtration, as described above. The enriched suspension was then incubated with 10 µM of CMFDA for 15 min at 37 °C. After incubation, 1 mL of PBS was added, and two washing steps were performed with 2 mL of PBS by centrifugation at 10,000 × g for 10 min. The final pellet was suspended in 100 µL of IMDM medium and then added to a new flask of HL-60 cells at a concentration of 2.10^5^ cells/mL^31^.

Third, three days post-infection, CMFDA-labelled HL-60 NY18 or HL-60 cells (1.10^6^ cells) were harvested by centrifugation at 500 × *g* for 5 min and washed with 1 mL of binding buffer A. The unfixed cells were then incubated with the conjugated aptamers at 23 °C for 30 min under agitation at 650 rpm. In total, four washing steps were performed by centrifugation at 500 × *g* for 3 min with 500 µL of binding buffer A.

Then, the final pellet was suspended in 200 µL of binding buffer A, before being analyzed on BD FACSCanto II (BioScience) with FlowJo™ software (version 10.8.1). The samples were tested in duplicates and the experiments were performed independently in triplicates. The negative control, corresponding to streptavidin AF-647 nm, was used and prepared in the same conditions as aptamers. The median fluorescence intensity (MFI) of scramble sequence was subtracted from the MFI of each sequence to eliminate non-specific background. Finally, Kd (constant dissociation) and Bmax (maximum of fluorescence intensity) values were determined by a non-linear regression analysis using GraphPadPrism 9® software.

### Study of aptamer binding to HL-60 NY18 or HL-60 cell lysates by fluorescence microscopy

The binding of aptamers was tested against HL-60 or HL-60 NY18 cell lysates. To obtain the lysates, 1.10^7^ HL-60 or HL-60 NY18 cells were harvested by centrifugation at 500 × g for 10 min and washed with 1 mL of PBS. The cells were suspended in 10 mL of PBS and then lysed using a Dounce homogenizer (20 round trip). After centrifugation at 750 × g for 5 min, the supernatant was filtered with 2 µm filter. The final lysate was recovered after centrifugation at 10,000 × *g* for 10 min. In the case of HL-60 NY18 cells, the bacteria were labelled with CMFDA, as described above. The conjugated aptamers (10 nM) were then incubated with the cell lysates for 30 min at 23 °C at 650 rpm. After incubation, four washes were performed with 500 µL of binding buffer A, by centrifugation at 10,000 × *g* for 3 min. The final pellet was resuspended in 100 µL of binding buffer A and this suspension was cytocentrifuged on a microscopic slide, mounted in Prolong Antifade Diamond Mounting containing DAPI (Thermo Fisher Scientific, Waltham, USA). The slides were examined with immersion oil using a Leica DMi8 confocal microscope. The experiment was performed in triplicates.

### Study of aptamer binding to HL-60 or HL-60 NY18 cells by fluorescence microscopy

In a first experiment, the binding of the conjugated aptamers was tested against 1.10^6^ CMFDA-labelled HL-60 NY18 cells, harvested after three days of infection or 1.10^6^ HL-60 cells. The unfixed cells were incubated with 50 nM of the conjugated aptamers at 23 °C for 30 min at 650 rpm. After incubation, four washes were performed with 500 µL of binding buffer A, by centrifugation at 500 × g for 3 min. The final pellet was resuspended in 100 µL of binding buffer A and this suspension was cytocentrifuged on a microscopic slide and prepared for confocal microscopy, as described above. Each experiment was performed in triplicates.

In a second experiment, the conjugated aptamers (50 nM) were incubated with 1.10^6^ CMFDA-labelled HL-60 NY18 cells, after different times of infection (12 h, 24 h, 48 h and 72 h post-infection), according to the protocol described above.

In a third experiment, the binding of the conjugated aptamers (50 nM) was tested against 1.10^6^ CMFDA-labelled HL-60 NY18 cells at 4 °C. Three days post-infection, the infected cells were harvested, maintained for 1 h at 4 °C and incubated with the conjugated aptamers for 30 min at 4 °C at 650 rpm. The four washes were also performed at 4 °C and the microscopic slide was prepared, as described above.

The average fluorescence intensity of each sequence was calculated with the ImageJ software. The outline of each cell was manually selected (region of interest), as well as a background region. The average signal intensity (red channel) of the background region of interest was subtracted from the average signal strength of the region of interest. Thirty cells were analyzed for one experiment. In total, three experiments were performed independently (90 cells analyzed in total). The colocalization between the red pixels (conjugated aptamer) and the green pixels (*A. phagocytophilum*) was estimated with the Pearson correlation coefficient (PCC), obtained using the JacoP Plugin (ImageJ software)^29^. For this, individual cells were selected, and a composite image (red and green) was created by JacoP Plugin to estimate PCC. No threshold was applied. Ten cells were analyzed for one experiment, the average of PCC was then calculated for the three experiments, performed independently.

### Impact of trypsin pre-treatment on aptamer binding

After three days of infection, 1.10^6^ CMFDA-labelled HL-60 NY18 cells were harvested at 500 × *g* for 10 min and washed with 1 mL of PBS buffer. The cell pellet was incubated with 500 µL of 1 X trypsin and 0.53 mM EDTA (Thermo Fisher Scientific, Waltham, USA) at 37 °C for 10 min or 30 min. After incubation, trypsin was inactivated by adding 500 µL of fetal calf serum. After centrifugation at 500 × *g* for 10 min, the cell pellet was washed with 500 µL of PBS buffer and used in the aptamer binding assay, as described above for fluorescence microscopy. The experiment was performed in triplicates.

### Study of aptamer binding in tick cells by fluorescence microscopy

Finally, the binding of aptamers was tested against ISE6 cells, ISE6 cells infected with *A. phagocytophilum* NY18 strain (ISE6 NY18) or ISE6 cells infected with *A. phagocytophilum* NV2Os strain (ISE6 NV2Os). The cells were harvested after centrifugation at 500 × g for 10 min and washed with 1 mL of PBS. The conjugated aptamers (50 nM) were incubated with 1.10^6^ unfixed cells for 30 min at 23 °C at 650 rpm. Four washing steps were performed with 500 µL of the binding buffer A, and the binding was assessed by confocal microscopy, as described above. The experiment was performed in triplicates.

### Supplementary Information

Supplementary Figures.

Supplementary Tables.

## Data Availability

Sequence data that support the findings of this study have been deposited in the European Nucleotide Archive with the primary accession code PRJEB62495.
