# Supplementary material for: Aptamer selection against cell extracts containing the zoonotic obligate intracellular bacterium, Anaplasma phagocytophilum
Source: Sci Rep. 2024 Jan 30;14:2465. doi: 10.1038/s41598-024-52808-8 (PMC10828505; doi:10.1038/s41598-024-52808-8)
Supplement: Supplementary file 1 — Supplementary Figures. [file 41598_2024_52808_MOESM1_ESM.docx]

***Supplementary Material***

**Aptamer selection against cell extracts containing the zoonotic obligate intracellular bacterium, *Anaplasma phagocytophilum***

**Lisa Lucie Le Dortz^1*^, Clotilde Rouxel^1^, Quentin Leroy^1^, Frédéric Ducongé^2^, Henri-Jean Boulouis^1^, Nadia Haddad^1^, Pierre Lucien Deshuillers^1^, Anne-Claire Lagrée^1^**

1. **Supplementary Data**
2. **Supplementary Figures and Tables**

# Supplementary Figures

#
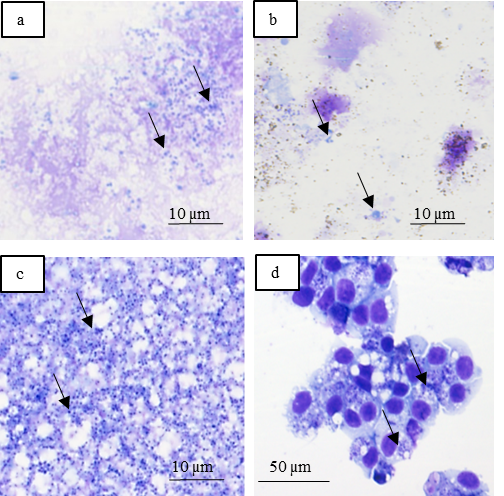


**Supplementary Fig. S1.** Representative image of Hemacolor® stained *A. phagocytophilum* enriched suspension, prepared using syringe lysis and differential centrifugation **(a)**, rock tumbler grit and differential centrifugation **(b)** or Dounce homogenizer and differential centrifugation **(c)**. Representative image of Hemacolor® stained HL-60 NY18 cells, after three days of infection with the *A. phagocytophilum* enriched suspension, prepared with a Dounce homogenizer. Hemacolor® stained DNA with azur B solution and cationic proteins with eosin. Cytoplasms have a grey color while nuclei are stained in purple. *A. phagocytophilum* is stained in dark purple (arrows) **(d)**.


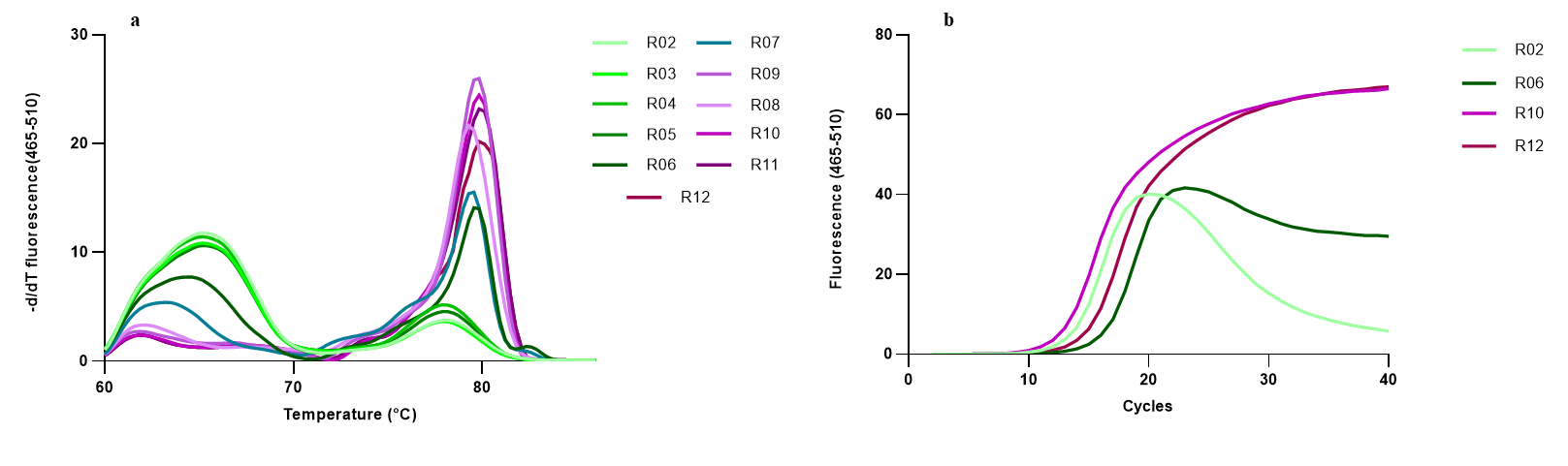


**Supplementary Fig. S2.** Monitoring of the SELEX by qPCR. **a:** Amplification curves obtained after qPCR of the final ssDNA pool (R02, R06, R10 and R12). **b:** Melting curves obtained after qPCR of the final ssDNA pool, from R02 to R12. A Tm around 64 °C corresponds to heteroduplexes, unstable compounds. A Tm around 79 °C indicates the amplification of homoduplexes, or stable compounds.


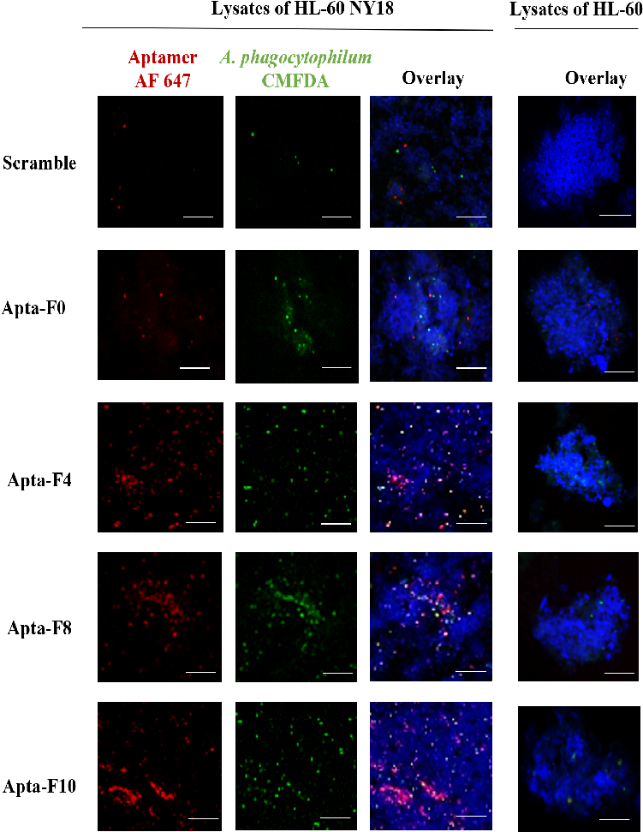


**Supplementary Fig. S3.** Confocal microscopy binding study against HL-60 and HL-60 NY18 cell lysates. Nuclei were labelled with DAPI (blue), and biotinylated aptamers were pre-conjugated with streptavidin-AF-647 nm (red). *A. phagocytophilum* NY18 strain was labelled with CMFDA (green). Scale bars represents 10 µm.


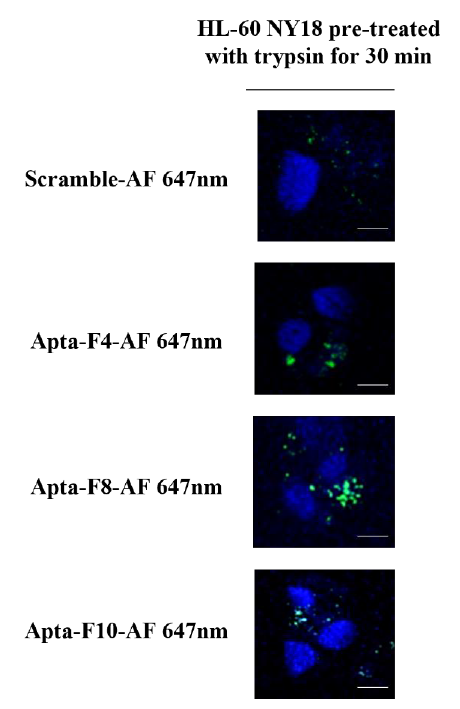

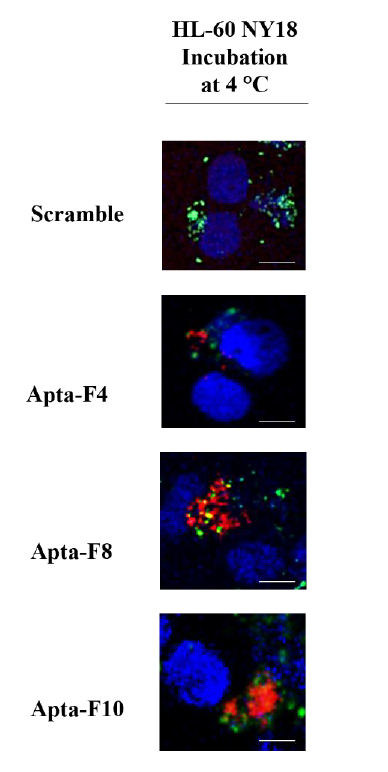
**Supplementary Fig. S4.** Confocal microscopy binding studies against HL-60 NY18 cells at 4°C. After three days of infection, the unfixed cells were incubated with aptamers at 4°C. Nuclei were labelled with DAPI (blue) and biotinylated aptamers were pre-conjugated with streptavidin-AF-647 nm (red). A. phagocytophilum NY18 strain was labelled with CMFDA (green). Scale bars represents 10 µm.

**Supplementary Fig. S5.** Confocal microscopy binding studies against HL-60 NY18 pre-treated with trypsin. After three days of infection, the cells were pre-treated with trypsin for 30 min. After inactivation, aptamers-647 nm were incubated with the unfixed cells. Nuclei were labelled with DAPI (blue) and aptamers-647 nm in red. A. phagocytophilum NY18 strain was labelled with CMFDA (green). Scale bars represents 10 µm.

# Supplementary Tables

**Supplementary Table S1.** Sequences clustered into families and their frequencies during SELEX. The frequency of each sequence at different rounds is given as a percentage of the library. This table shows only those sequences containing at least one sequence with a frequency greater than 0.02 % in a round. Sequences were clustered into families using a Levenshtein distance of 8. Sequences evaluated in binding are underlined in yellow.

**Supplementary Table S2.** Evolution of different families during SELEX. The frequency of each family at different rounds is presented in percentage of the library. Families are composed of sequences clustered using a Levenshtein distance of 8. The sequences that are provided in left correspond to the most abundant of the family. This table presents only the families which contain at least one sequence with a frequency higher than 0.02 % in one round. The sequences evaluated in binding are underlined in yellow.

**Supplementary Table S3**. Multiple alignment of the most enriched families. Families assessed in binding are highlighted in yellow and their similarity to other families is boxed.
